# Retraction: Study on the Effect of Oral Administration of Bacteriophages in Charles Foster Rats with Special Reference to Immunological and Adverse Effects

**DOI:** 10.3389/fphar.2021.824722

**Published:** 2021-12-22

**Affiliations:** Frontiers Media SA, Lausanne, Switzerland

**Keywords:** oral, adverse effect, cytokines, klebsiella peneumoniae, bacteriophage therapy

The journal retracts the 12 April 2021 article cited above for the following reasons.

Following publication, concerns were raised regarding the integrity of the methodology used in the study. The authors failed to provide a satisfactory explanation during the investigation, which was conducted in accordance with Frontiers’ policies.

This retraction was approved by the Chief Editors of Frontiers in Pharmacology and the Chief Executive Editor of Frontiers. The authors did agree to this retraction.

